# Report from the Medical Library Association’s InSight Initiative Summit 3: Bridge Building: What Bridges to Build and How

**DOI:** 10.5195/jmla.2020.894

**Published:** 2020-04-01

**Authors:** Katherine G. Akers

## Abstract

At the Medical Library Association’s Insight Initiative Summit 3, held June 12–13, 2019, academic and hospital librarians joined with publishing industry partners to identify vexing problems in publishing and accessing health sciences information. Through a mixture of panel discussions with health sciences faculty, librarians, and information providers; small-group problem-solving exercises; and large-group consensus-building activities, the summit program invited participants to appreciate each other’s viewpoints and propose a collaborative project leading to tangible outcomes that could ultimately benefit end users. Several vexing problems were identified, including poor communication and mistrust between librarians and publishers, complexities in product pricing structures and licenses, and users’ difficulties in accessing and using vetted information resources. However, librarians and publishers agreed that building a better shared understanding of users’ needs and behavior would be the most useful bridge toward regaining trust, establishing more effective partnerships, and designing and delivering quality information resources that are easily accessible and maximally useful to health sciences researchers, educators, clinicians, and students.

The Medical Library Association (MLA) InSight Initiative Summit 3, held June 12–13, 2019, in Chicago, Illinois, brought together library leaders and publishing industry partners to engage in high-level, high-value dialogue on issues of common interest that impact the health information profession. Drawing upon advances made by Summit 1 and Summit 2 participants in recognizing the evolving needs of health sciences information users in a disruptive era, participants of Summit 3, “Bridge Building: What Bridges to Build and How,” identified the most vexing problems in publishing, delivering and accessing quality health sciences information, and built bridges between librarians and information providers leading toward tangible outcomes that could benefit users. The program included a mixture of panel discussions with health sciences faculty, academic and hospital librarians, and different types of information providers; small-group problem-solving exercises; and large-group consensus-building activities.

## WELCOME

Daniel J. Doody, summit facilitator, and Gerald (Jerry) Perry, AHIP, FMLA, liaison from the InSight Initiative Task Force, welcomed Summit 3 participants. In this time when our future as providers of vetted medical information is uncertain, with both libraries and the publishing industry under threat, Doody said we must move past treating each other as the enemy, as cooler, smarter heads would prevail. He thanked participating organizations and the program committee for supporting and planning the summit.

Doody told participants that they were in a privileged position and urged them to treat the summit as a retreat. He asked them to be “all in”—disengaged from their devices—and show respect to other participants, speak truthfully, and listen attentively. He asked participants to commit time and mindshare to collaborating on future tangible outcomes agreed upon by the group and to engage in Summit 3 discussion with consideration of how they could be built upon in Summit 4.

Doody indicated that although financial issues were “off the table” in Summits 1 and 2 to reduce tension and promote good will, financial issues could be part of the discussion in Summit 3. Participants were given written guidelines for talking about product pricing and licensing agreements to comply with legal regulations against collusion and to continue to foster good will. These guidelines included anonymizing references to specific vendors, products, and institutions; not discussing specific product prices or pricing structures; and not describing specific price negotiations between institutions and information providers.

Perry described the InSight Initiative as being born from an ethos of mutuality in recognition of long-standing commonalties between librarians and publishing organizations. Both parties care about information access and use but operate in persistent economic frameworks and perceive tensions between their communities. When Perry started working as a library administrator at the University of Arizona, he had robust conversations with Association of Research Libraries leaders regarding library philosophies about information access and working with information providers. He discerned that many thought leaders are skeptical and somewhat distressed about commercial publishing organizations with whom libraries work. Perry said we cannot afford to operate this way anymore and questioned why we do not trust each other and how we can rebuild trust, especially in this time when both communities are faced with existential risks. He posited that real bridges are the best way to ensure mutual relevance. Perry hoped that we can grow and sustain mutual appreciation of each other’s contributions while also recognizing risks and benefits. Although publishers and libraries will likely continue to exist, Perry said he wonders about our future roles and value proposition to users. Doody thanked Perry for his challenging words of welcome.

## PANEL 1: CHALLENGES AND OPPORTUNITIES FACING USERS

Doody explained that the most valuable component of Summit 2 was a panel of health care professionals with impressive biographies who spoke about how, where, and on what devices they discover, access, and consume information in their fields [1] and who returned to MLA ’19 to give the John P. McGovern Award Lecture. Due to the insights gleaned from that panel, Summit 3 activities commenced with another user panel consisting of clinical and basic science faculty members from leading Chicago-area institutions. All panel members spoke of “wearing different hats” as researchers, clinicians, educators, and administrators and agreed that one of their most vexing problems was the existence of too much information in their fields.

### Rosalyn P. Vellurattil, Assistant Dean for Academic Affairs, University of Illinois–Chicago

Rosalyn P. Vellurattil is a clinical professor and administrator primarily involved in curricular and faculty development. She opened the panel by sharing a success story of enlisting a librarian to help conduct focus groups on students’ impressions of faculty members’ cultural competence. She described the experience as “eye-opening” and was impressed by the librarian’s expertise in using databases to discover relevant information, which helped the team work more efficiently. The resulting project was published as an article in the *American Journal of Pharmaceutical Education* [2], which received a prestigious award. To keep up to date in her field, Vellurattil takes advantage of continuing education opportunities, primarily during national pharmacy conferences but also through webinars. She receives electronic tables of contents (eTOCs) from her favorite journals and bookmarks or prints articles for later reading. Her biggest frustrations in keeping up to date are her lack of time and inability to efficiently conduct literature searches. Vellurattil shared several ideas for services or resources that could be provided by publishers, including direct solicitations for authors to write about identified gaps in the literature; a resource like UpToDate geared toward educators, which could be developed collaboratively by publishers and librarians; and new resources that could “hit health professionals on the go,” such as brief sound bites.

### Raj C. Shah, Associate Professor of Family Medicine, Rush University

Raj C. Shah described eight skills that medical students at his institution are expected to gain during their program. Of these, he focused on the roles of scholar and educator and how they interact with medical knowledge. As scholars, medical professionals must keep abreast of new findings and build upon existing knowledge to create new knowledge. Shah described the usefulness of PubMed alerts for keeping up to date but noted that relying solely on this approach misses grey literature and news articles. He described using the services of a librarian who identified influential articles to help him decide whether to continue or alter one of his research studies. He also acknowledged the National Institutes of Health’s mission of public access but noted the pressure of working with journals that charge author fees and the difficulty in paying for open access (OA). As educators, medical professionals must be able to impart research skills to others. Shah described working with librarians on educational projects such as creating career development plans for new faculty, including how to use the library more efficiently, and teaching new ideas and concepts, such as precision medicine, to medical students.

### Nicola Orlov, Assistant Professor of Pediatrics, University of Chicago

Nicola Orlov is a pediatrician hospitalist, director of a residency program, and mother. She described herself as “living two lives” at work: providing clinical services and mentoring. Orlov finds medical information through a myriad of approaches: using Twitter to identify new, noteworthy journal articles; posing questions to a clinical librarian rather than performing PubMed searches herself; using UpToDate; participating in email discussion lists; scanning eTOCs from her favorite journals; attending conferences; and using Doximity, a medical networking site. She finds time to consume information early in the morning with coffee before the rest of her household wakes, during morning report and journal club, while walking the halls of the hospital, and at her desk during dedicated scholarship time. She is frustrated by the size of the body of medical information and is overwhelmed by the volume of PubMed search results, which prompts her to rely on a librarian. She feels inundated with emails, often struggles to access institutionally licensed information resources, and has not yet figured out how to deal with non-peer-reviewed medical information discussed by her friends and family members.

### Michael Calik, Assistant Professor of Biobehavioral Health Science, University of Illinois–Chicago

Michael Calik is a basic neuroscientist who uses animal models in his research. To discover relevant journal articles, he relies on Google Scholar recommendations based on his published work, which he feels are very accurate, and PubMed alerts. Being an F1000 Faculty Member, he reads others’ work and writes his own article recommendations, and he admitted surprise upon later learning that F1000 was a paid resource. He follows select journals and scans the eTOCs of new issues. To facilitate accessing journal articles relevant to his work, Calik created a personal storage system on his computer, in which he saves portable document format files (PDFs) in specific folders by category and locates articles at a later date by performing a Windows search. If he cannot find an article stored on his computer, he performs a PubMed search.

He likes his library’s PubMed LinkOut, which he says works 80% of the time; the other 20% of the time, he follows his library’s links to arrive at a prepopulated interlibrary loan (ILL) request form. Prodded by the nagging feeling that he is missing something, he sometimes performs Google Scholar searches to catch articles that might be missed in a PubMed search. His biggest frustration is the sheer amount of information being published, such that he even checks his article alerts while walking to and from his car at work. He does not see a solution to this problem. Rather, he views the use of his custom-built system for storing and accessing journal articles as necessary for being a competitive researcher and “keeping sane,” and he feels blessed by the resources provided by his university library. Calik noted that he loves when publishers prepare PowerPoint slides containing high-resolution images of results reported in journal articles with accompanying citations, which are helpful in his teaching.

### Peggy Mason, Professor of Neurobiology, University of Chicago

Peggy Mason is another basic neuroscientist who performs research using animal models. She reflected on how much the information landscape has changed since she began conducting research. She said that teaching a massive open online course (MOOC) exposed her to “true diversity” and revealed the constraints in accessing information faced by people living in different countries. As a result of this experience, Mason asserted, “My message is open access, open access, open access, open access; that’s all I have to say.” Although she understands the economic issues with OA, she is an “OA convert” because paid access does not work for her or her students. That she gives OA articles more weight in her teaching due to convenience rather than an intellectual choice is “a bad thing,” and she said she did not have a lot of respect for scientific publishing at the present moment. She described using Twitter to stay abreast of new research findings but is concerned by the fact that not everyone can access the articles being tweeted.

### Ryan Crews, Assistant Professor of Podiatric Surgery and Applied Biomechanics, Rosalind Franklin University of Medicine and Science

Ryan Crews is a clinical research scientist. Regarding his teaching duties, Crews noted that students must be able to seek out and obtain medical literature and are required to take an “Understanding and Implementing Clinical Research” course in their first year, in which a librarian gives a one-hour lecture on searching PubMed. As a PubMed user, Crews likes using Medical Subject Headings (MeSH) terms, publication year limits, and LinkOuts to institutional subscriptions. He is a fairly active user of ResearchGate, which he uses to learn about new articles published in his social network and see how others interact with his work. He uses Mendeley and Academia.edu for similar purposes and finds LinkedIn helpful for interacting with industry connections. He uses Google Scholar to track his publication metrics and receive article recommendations, and he uses journal impact factors and SCImago journal rank indicators to gauge the quality of unfamiliar journals. Crews is concerned with the number of predatory publishers in existence and frequently receives spam email solicitations from journals. To vet potentially predatory journals, he relies on Beall’s list, which is now defunct; the Directory of Open Access Journals (DOAJ); and Google searches to gauge public sentiment about particular journals.

### Susan Buchholz, Professor and Associate Chairperson, Adult Health and Gerontological Nursing, Rush University College of Nursing

Susan Buchholz focuses on nursing leadership and engages in administrative activities, teaching, research, service, and nursing practice. She keeps up to date in her area by checking email alerts received from associations, journals, the Centers for Disease Control and Prevention (CDC), and PubMed; following blogs; and reading op-ed articles. Whereas she used to scan article titles in the eTOCs of journals, she now scans for recognizable author names. She still uses books but also searches PubMed and other article databases and consults UpToDate. She is a frequent library user who regularly calls the library and works with librarians, including asking them for assistance with literature searches. She said that most resources that she wants are available through the library but otherwise are available through ILL. She uses a myriad of online resources in her teaching and coteaches a literature synthesis course with a librarian. On her information wish list are personalized, scheduled daily email briefings containing summaries of and links to articles as well as a way to more easily manage a bibliography of her authored works without having to separately keep up ResearchGate and ORCID accounts.

### Question-and-answer session

A librarian noted that many panelists mentioned that waiting one to two days to receive an article through ILL is too long, whereas some found the wait acceptable. One panelist reiterated her opinion that one to two days is too long, saying that if an article is not immediately available, it ceases to exist in her mind. Another panelist said that, while writing, he surmises essential information from abstracts and then waits for the full-text articles to arrive to fill in the details. A third panelist said that completing ILL forms is annoying, but that the articles arrive pretty quickly.

A publisher asked if panelists used preprint servers. One panelist said she always posts her manuscripts on bioRxiv but called it a “black hole of publishing.” Although preprint servers were developed to foster public commenting on manuscripts before they undergo formal peer review and publication, she finds that no one comments on manuscripts and that her work receives more attention on her blog. Another panelist likes preprint servers because they provide digital object identifiers (DOIs) for articles in preparation, which can serve as evidence of productivity for grant applications. Finally, a panelist said he never looks at preprints because he was taught to believe that scholars should only consider peer-reviewed material. Although he thinks preprint servers are a way to informally publish negative findings, he also described them as “black hole[s] of stuff that didn’t work,” with no one seeking their content.

## SMALL GROUP EXERCISE #1

Participants gathered in four small groups with roughly equal representation by librarians and publishing industry representatives to discuss vexing problems from the user’s perspective that could be solved collaboratively. Problems commonly identified by groups included bias in peer review and publishing; slowness of the peer-review and publication processes that delays scientific and medical progress; too many unstandardized and inoperable platforms for information discovery; paywalls and copyright being obstacles to teaching and research, causing users to select information based on its ease of access rather than its quality; insufficient ways for users to filter or tailor information to meet their needs; and users’ poor search skills and lack of awareness of how to access information through licensed avenues.

## PANEL 2: CHALLENGES AND OPPORTUNITIES FACING MEDICAL LIBRARIANS

In the next panel, leaders in different types of libraries—general academic, hospital, and academic health sciences—spoke about challenges to and opportunities in meeting the information needs of library users.

### Stephen Bosch, Content and Collections Librarian, University of Arizona

Stephen Bosch, a regular author on the topic of academic serial prices [3, 4], spoke of the “the perfect storm” hitting the economy of scholarly and professional publishing, arising from steady increases in the cost of information, stagnant library funding, and changing user expectations for access. He reported that higher education expenditures for libraries have dropped from 3% to 2% over the last 20 years, but the cost of serials has increased by ~6% annually over recent years, which is far faster than the rise in university tuition cost and National Science Foundation (NSF) research expenditures. Thus, the “serials crisis” has not gone away, and libraries are being left behind due to a steady erosion in purchasing power.

Bosch argued that the academy needs to engage with and take ownership of the problem of library funding. If the academy wishes to sustain the current system by which faculty are rewarded for publishing in high-profile journals, it must be willing to fund that system. It must also accept that the current state is not sustainable.

Bosch proposed that if technology has driven this problem by expanding the breadth of the digital scholarly record and increasing user expectations for access, it could also be used to solve the problem. Bosch predicted that artificial intelligence (AI) will impact analytics, assessment, peer review, clinical tools, and “things we haven’t even imagined yet.” In particular, AI could be used to summarize and help users sift through information. However, we must keep in mind that using big data to make business decisions occurs to the detriment of user privacy.

### Lisa Carter, Director of Library Services, Hartford Healthcare

Lisa Carter works for a hospital system that consists of ten sites with teaching, community, or specialty purposes that are evolving into institutes with unique focuses. Based on her experience, she described several challenges facing hospital libraries and suggested ways that publishers could help.

#### Hospital mergers

When hospitals merge, reporting structures are in flux, new administrators may give libraries low priority due to their non-revenue-generating status, Internet protocol (IP) address ranges may be shared, and subscriptions may need to be consolidated. Thus, publishers could allow a grace period while hospital systems restructure.

#### Pricing structures

As health care providers at different hospital sites with different missions may not need or use the same resources, publishers could allow more flexibility in site licenses. Carter also recommended setting stricter rules for how individual users are counted (e.g., are rotating physicians counted twice?), more clearly defining hospital “tiers,” and providing shorter licensing periods to allow libraries to “pay as you go.”

#### Vendor representatives and technical support

Outgoing representatives could introduce incoming representatives to librarians, and human interaction should be given preference over trouble-ticking systems. Carter expressed displeasure with representatives visiting her hospital without her knowledge and recommended that physicians be recruited to help give product demonstrations, as “doctors do demos best.”

#### E-textbooks

As e-textbooks are demanded only by students and only if they are required for a course, publishers could allow more flexibility in concurrent licenses—perhaps on a semester basis—and connect with medical educators to determine which textbooks are most desired.

#### Usage statistics

Carter described a disconnect between “what is counted” and “what is useful.” She suggested that publishers allow self-retrieval of usage statistics, the option of an “exportable report” rather than “sheets of data,” and accessibility to a representative who can explain usage reports when the numbers do not add up.

Carter concluded by telling publishers that “we’re both passionate about your products. We’re on the same team, let’s work together.”

### Susan K. Kendall, AHIP, Coordinator for Health Sciences and Copyright Librarian, Libraries, Michigan State University

In light of rapid changes in medical education and rising student debt, Susan K. Kendall, AHIP, described an opportunity to design and deliver information resources that support medical education as it is envisioned and implemented in the twenty-first century [5], with less reliance on costly textbooks and greater employment of active learning and flipped classroom models. What this means is that educators must be able to “mix and match” content from multiple sources to create custom curriculum materials, and libraries, instead of students, will pay for information resources.

As support for this line of thinking, Kendall described a recent “Turn Med Ed on Its Head” challenge by the American Medical Association, in which the winning students proposed an online national exchange that is “equal parts information repository, social network and learning management system—where medical schools will publish their curricular materials as free, open-access content for use by educators and learners” [6]. She also drew attention to a question posted on the Student Doctor Network about whether it is possible to avoid using textbooks in medical school, to which respondents recommending using lecture notes, Google, or YouTube instead of textbooks [7].

Kendall expressed frustrations that the information resources available to libraries are not designed to meet the changing needs of medical education. She suggested that publishers could price e-textbooks more affordably for libraries as opposed to individual users and could design resources and licenses that allowed “remixing” [8] so that individual parts (e.g., chapters, sections, images, videos) and their copyrighted information can be pulled into custom curricula in course management systems.

As an example, her university’s College of Human Medicine deployed a new shared discovery curriculum system called “Just in Time Medicine” [9], in which students can log in to access learning modules that link out to videos, textbook chapters, and faculty-written materials containing textbook images. As traditional licenses do not allow materials to be incorporated into derivative works, her library negotiated with information providers to include an addendum in their licenses stating that they could use copyright-protected images in presentations and lectures posted in password-protected systems for educational purposes. Furthermore, librarians created guides to inform educators about image use permissions [10] and to create stable links to e-books [11] for each publisher. They estimated that this approach prevented the need for first-year veterinary students to purchase 26 textbooks, saving $2,900 per student.

To facilitate similar approaches by other institutions, Kendall asked whether publishers and librarians could collaboratively write licenses in which “authorized usage” allows remixing content for educational purposes and could develop standards for linking to specific content.

### Question-and-answer session

A librarian questioned whether the notion that open educational resources (OERs) are untrustworthy is a myth. Kendall answered that OERs are still in their infancy but are desired by medical faculty. Another librarian asked how library users—from reader and author perspectives—feel about OA. Carter answered that readers get annoyed with needing to go through a system to access journal articles and would prefer accessing articles directly. Kendall agreed that readers prefer OA articles because they are easily accessible and free but said that faculty who want to engage in OA publishing do not know who should be responsible for paying article processing charges and need applicable funding. She felt that users do not have major concerns about the quality of OA articles, as both OA and subscription journals have wide ranges in quality. A publisher asked for panelists’ thoughts on the “Read and Publish” model, in which the price of a journal depends on how frequently an institution’s authors publish in that journal. Bosch stated that the OA2020 and Plan S initiatives are not going to solve the problem, as they do not serve as equitable solutions for covering the costs of scholarly publishing.

A publisher asked Bosch about which information-seeking behaviors would be best served by AI. Bosch replied that AI is good for processing large amounts of information quickly—faster than humans. He said that AI could aid in summarizing the literature and make more sense out of analytics, predicting that AI “will allow us to do our jobs better, not take our jobs.”

Regarding analytics, a librarian expressed worry about user privacy issues, as publishers are collecting more data on product usage but might not be transparent about their use of that data. This librarian was concerned about monetizing data that should belong to the user or their institution by selling the information back to the institution in the form of analytics. They questioned whether there is “any way out of this morass,” because collecting user information is necessary for creating personalized recommendations. Bosch replied that higher education is a competitive environment and that advances in this direction are inevitable and do not necessarily need to be halted. Carter agreed that data on specific users can be valuable, saying that she needs to know who is using her library’s resources to demonstrate their value to administrators.

## SMALL GROUP EXERCISE #2

In small groups, participants were asked to think back to the vexing problems that were identified in the user panel, reflect on issues raised by the librarian panel, and brainstorm tangible outcomes and potential next steps. The following vexing problems generated the most compelling ideas for potential tangible outcomes and next steps.

### Lack of communication

Vendors do not know what users or librarians want, and librarians do not know what they can ask for. Each library and publisher could have a dedicated contact person to ask and answer questions about products, such as whether a product license allows specific components to be used in a MOOC course pack.

### Variability and lack of transparency in vendor pricing models

Publishers and librarians could form a collaborative working group to identify issues, explore different pricing models, and work toward optimizing and standardizing pricing models across products and vendors.

### Complexity of product licenses

Publishers and librarians could create avenues for negotiating product licenses more simply and proactively, such as by establishing negotiation ground rules or standardizing licenses so they could be reviewed more quickly. A next step could be to explore Shared E-Resource Understanding (SERU) best practices [12].

#### Ever-increasing prices for libraries

Publishers could better explain their pricing models to librarians and be more transparent about reasons for price increases. Libraries could place greater effort on advocating for more funding from their institutions.

#### Underuse of quality resources

Users opt for ease of access at the risk of quality. Librarians and publishers could obtain a better understanding of and embrace user behaviors, such as through ethnographic or mixed methods studies; align avenues of access with actual user workflows; and create educational materials to improve user skills in evaluating information.

Rich Lampert, summit facilitator, led a discussion among all participants to narrow down the vexing problems most in need of a solution. The discussion touched upon many problems, including the non-sustainability of the scholarly publishing business model; lack of communication between publishers and librarians; complexity of licensing terms, especially for hospitals; poor understanding of user needs and behaviors; underuse of quality resources when users opt for convenience; and product interfaces that are not standardized and not designed to meet the needs of novice and expert searchers. Reminding participants to keep the end user in mind, Lampert asked participants which problems seemed most vexing. Many participants indicated “lack of communication” or “underuse of quality resources,” some indicated “opaque business models,” and few indicated “complexity of licensing terms.”

## PANEL 3: CHALLENGES AND OPPORTUNITIES FACING INFORMATION PROVIDERS

The third panel featured senior executives from three participating organizations representing different types of information providers: a mainstream publisher, a professional society publisher, and an information aggregator.

### Susan Haering, Director, NEJM Group Licensing

In her talk, “What I Wish You Knew,” Susan Haering shared challenges and opportunities from a publisher’s perspective. She said that subscription renewals can be either a time of lengthy negotiations around price or an opportunity to engage around value. Publishers need to better understand how librarians define value, which may include not only cost per use, but also impact. For example, an article published in the *New England Journal of Medicine* (NEJM) can be a major career boost for an author. In addition, publishers with small support staffs have challenges handling a large volume of customized service requests, such as running usage reports for individual accounts or making Internet protocol (IP) address changes. Therefore, publishers need to find a better way to help librarians automate reports and manage IP addresses across multiple publisher systems. Accurate IP address management is especially important because both sides want to know that the IP addresses associated with a license are correct and that usage reports accurately reflect user activity.

Open access is another issue closely watched by NEJM Group and other society publishers. Haering explained that NEJM’s editorial team adds a great amount of value to content, including hundreds of hours spent on curation, review, verification of statistical analyses, and removal of hyperbole before an accepted manuscript is published. She said that being good and innovative is expensive and requires the work of a large editorial team and technological infrastructure to support new kinds of content and functionality.

Haering said that NEJM Group seeks opportunities for better engagement with librarians and their patrons. She asked librarians in the audience to “help us help you.” How can publishers help librarians educate readers and authors about their products? How can publishers help librarians better understand how products are used by library users, and how can librarians help publishers better understand user information needs? She concluded by reminding summit participants that “we’re all in this together.”

### Rose Sokol-Chang, Journal Publisher, American Psychological Association

Rose Sokol-Chang introduced the American Psychological Association (APA) as a nonprofit publisher that engages with the communities they serve, works with both researchers and practitioners, and strives to be a good fit to everyone in their community. She highlighted three issues that are important to the APA.

#### Access

The APA wants to make it easy for users to access content regardless of their institutions or countries. However, they face challenges in managing authentication across systems and facilitating content discovery. They want to build an OA model that works for everyone but are unsure of how to engage in “Read and Publish” deals [3] without publishing a large number of journals.

#### Open science and methodology

The APA wants to ensure that they publish sound research with clearly described methods and promote the sharing of research materials. However, they face challenges in offering incentives for authors to share research materials that are “beyond the article” (e.g., data sets), as most incentives are afforded by a researcher’s place of employment.

#### Impact

The APA wants the research they publish to reach and impact the communities that need it most. However, they face challenges in making specialized information accessible to laypeople and successfully measuring its impact.

Sokol-Chang asked how information providers and librarians can work together to promote efforts to keep science strong. She suggested that keeping a focus on our shared community while recognizing our unique contributions would be a good starting point.

### Steven Heffner, Vice President of Product Strategy, Wolters Kluwer Health Learning Research & Practice

Steven Heffner said that the most vexing problem for Wolters Kluwer, as a commercial publisher, is the lack of mutual trust between their organization and librarians. Perhaps counterintuitively, he said the fact that they “are out to make a buck” should be the very basis of librarians’ trust. That is, their intention to make money should be reassuring because “we prioritize our products to provide return on investment for end users.” He said that they are absolutely dedicated to open science and improving patient care and that they measure their success by their revenue.

He also expressed frustrations with encountering stiff resistance from librarians in providing information about their users, saying that they sometimes have to go around librarians to get the information they need, such as through focus groups, online user identification and analytics, and “spying” on users to identify their workflows and use cases.

Heffner said that because for-profit publishers are good at marketing, they can partner with librarians to increase awareness of their resources. Because they are good at sales, they can help librarians identify pools of funding from their institutions. They are also really good at product development. Thus, publishers and librarians want the same outcome for different reasons.

However, Heffner acknowledged that building trust is not easy and requires collaboration. He suggested that MLA could facilitate continuing education programs that could be attended by both librarians and publishers, such as sales training to increase product usage, as people from different sides sitting at same table—learning and experiencing together—could help build trust.

### Question-and-answer session

A librarian asked Heffner to elaborate on his statement about mutual distrust between publishers and librarians: librarians’ reasons for distrusting publishers have been extensively voiced, but why would publishers not trust librarians? Heffner explained that publishers distrust librarians because they do not help deploy user surveys, resist the collection of data from users, and are ideologically driven advocates of OA. Haering said that NEJM Group does not mistrust librarians, but they lack an understanding of who is using their products, which might be a result of a lack of communication. Doody said that when acting as a representative of Doody Enterprises, he never felt welcomed into libraries, was given a minimal amount of time with users, and was “painted as a villain.” He said, “A little welcome would go a long way.” A librarian countered that their distrust is not aimed exclusively at publishers; rather, librarians have a long history of distrusting government tracking of library records and are thus committed to protecting one’s freedom to access information.

A librarian said they understand the value added by NEJM Group, but that people who hold the purse strings in institutions do not care about the quality of information. A publisher in the audience concurred that people will choose “cheap over quality” until something bad happens. Haering said that when dealing with human lives, “we are going to throw lots of resources into delivering high-quality information.” Some publishers in the audience talked about how they must consider where quality counts the most, agreeing that copyediting individual articles does not add as much value as larger efforts to guard against scientific fraud or enhance discoverability and access.

Doody asked the panel members two questions: (1) what is one thing you want librarians to leave with that is “bridgeable,” and (2) what does a better ecosystem look like twelve months from now? Heffner replied that he wanted librarians to know that “we are very simple—you know what we want. We are not nefarious. We are selling in your community because we participate in same value chain as you.” He said publishers and librarians could achieve a healthier ecosystem if they mutually built a usage analytics data set that was open and transparent. Sokol-Chang agreed that “we need to share information about our users’ needs so that we can address them together.” Finally, Haering said she wanted librarians to know that “we are open to conversations and questions, we are human, and it’s okay to ask why we are doing things.” In twelve months, she wanted librarians to fully understand NEJM Group’s resources and be a mouthpiece to users, with publishers taking the responsibility to give librarians tools to get the word out.

## SMALL GROUP EXERCISE #3

Participants were asked to think back to the vexing problems that were identified in the user and librarian panels, reflect on issues raised by the information provider panel, and brainstorm tangible outcomes and potential next steps in small groups. The following vexing problems generated the most compelling ideas for potential tangible outcomes and next steps.

### Poor understanding of user information needs and behavior

Tangible outcomes could include getting information resources to users in the right place at the right time, providing publishers’ assistance in marketing resources and educating library users, and creating a publisher-librarian community for sharing user feedback. Next steps could include engaging in collaborative efforts to better understand user behavior (e.g., surveys), scheduling librarian-led webinars to help publishers better understand the needs of different types of users, obtaining analytics as evidence of user needs and behavior, and creating user and/or library advisory boards for specific publisher projects or products.

### Difficulty in using information resources

Because “faculty make decisions on what works, not what works best,” next steps could include collaborative efforts to standardize search interfaces, provide easier remote access and navigation to products, and test new product features.

### Lack of trust between publishers and librarians

Publishers undermine trust by using sales tactics, circumventing librarians and dealing directly with end users, and not respecting user privacy. A tangible outcome could be a “do’s and don’ts” document detailing best sales practices and describing tactics used in the past that eroded trust and led to repercussions. Next steps could include a collaboratively written manifesto or shared understanding of values, discussion about how vendors should contact librarians and end users (e.g., making sure the library is at the center of institutional negotiations), more open communication around each party’s motives, agreement on which analytics are most appropriate for understanding user needs and behavior, and knowledge-sharing through vendor-sponsored webinars about topics that may be unfamiliar to librarians (e.g., licensing constraints, the sales cycle/process). Existing MLA infrastructure could be used to disseminate resulting documents and to host webinars or other continuing education opportunities.

## SELECTION OF TANGIBLE OUTCOME PROJECT AND WORK PLAN

Kevin Baliozian, executive director of MLA, said it is great that librarians and publishers are talking about transparency, communication, and collaboration. However, his thoughts kept returning to the “elephant in the room.” When he conducted a quick poll, most summit participants—both librarians and publishers—agreed that the cost of education and health care in the United States is too high. However, only librarians agreed that the cost of publishing is too high. Baliozian said, “Until there is a feeling that there is a joint problem, it’s not going to be solved.” He wished for “a joint ‘aha’ moment that there is a problem—a macroproblem” in the cost of publishing, which he thinks would go a long way toward generating trust. Doody pointed out that the non-sustainability of the publishing business model had been a topic of summit conversations, indicating awareness of the issue on both sides.

Doody then steered the discussion toward understanding user behavior. A publisher reminded participants that although summit conversations focused on the divide between librarians and publishers, publishers are also very competitive with each other. “We don’t want a shared understanding of user behavior. We want a unique understanding of user behavior. We fight against and don’t trust each other.” A librarian concurred by saying that user data, in aggregate, would not be very useful to publishers, as each publisher wants a unique insight for monetary footing. After some cursory discussion of issues related to understanding the needs and behaviors of diverse users, Doody stated that participants should set some ground rules because data resulting from a study of user behavior would be entering a competitive arena.

Doody asked participants to vote for the most vexing problem identified in the summit. Most participants chose “lack of trust,” some chose “poor understanding of user behavior,” and none chose “difficulty in using information resources.” However, when participates voted again with respect to a potential project with a tangible outcome, most selected “poor understanding of user behavior,” with “lack of communication” a close second and “lack of trust” a distant third place. When Doody asked what a tangible, enduring outcome might look like, participants suggested written ground rules or best practices for collecting user information, a manifesto on agreed-upon values in regard to user needs, a forum in which librarians could communicate with publishers about user interface issues, a webinar or white paper on understanding user needs, the delivery of an InSight Initiative–branded curriculum on user behavior using existing MLA infrastructure, or a research project on information discovery from the user’s perspective.

Doody indicated that Summit 3 participants should return for Summit 4 as a retreat for producing a tangible outcome. In the meantime, he recommended that participants collaborate on performing a review of literature about user behavior, designing a survey to assess user needs or behavior, agreeing on ground rules for negotiations, planning a forum on improving user interfaces, creating an end-user advisory board, and defining the overall scope of the tangible outcome project.

## WRAP UP AND ANNOUNCEMENTS

Doody concluded the summit by soliciting three librarian and three participating organization representatives to serve on the Summit 4 Program Committee. He thanked the panelists, participants, program committee and facilitators, participating organizations, summit reporter, and MLA. He congratulated participants on deciding “which bridge to build” and what the outcomes will look like and asked them to talk about the summit with their colleagues and/or supervisors. Baliozian stated that participating organizations would be asked to provide feedback on the InSight Initiative funding model to help determine whether the initiative could continue sustainably after its pilot period, emphasizing that MLA wanted the initiative to be successful.

## 

**Katherine G. Akers, PhD**, katherine.akers@wayne.edu, https://orcid.org/0000-0002-4578-6575, Editor-in-Chief, *Journal of the Medical Library Association*, and Biomedical Research and Data Specialist, Shiffman Medical Library, Wayne State University, Detroit, MI

## SUGGESTED PRE-SUMMIT READING SELECTIONS

- Anderson E. Print to electronic: the library perspective. Publishing Res Q. 2016 Mar;32(1):1–8. DOI: http://dx.doi.org/10.1007/s12109-015-9440-5: An overview for publishers of key challenges facing libraries in the digital world. Describes library technical environments, licensing issues, pricing issues, content, and usage statistics. Concludes with a brief section on needed areas of collaboration between libraries and publishers.
- Bosch S, Albee B, Romaine S. Deal or no deal. Libr J. 2019;144(3):34–9. <https://www.libraryjournal.com/?detailStory=Deal-or-No-Deal-Periodicals-Price-Survey-2019&utm_source=Marketing&utm_medium=Email&utm_campaign=top5>: Extensive survey of journal subscription prices in all disciplines.
- Folan B. Librarians’ messages to publishers: turning research into practice. Insights. 2017;30(3):126–37. DOI: http://dx.doi.org/10.1629/uksg.390: Reports on a survey of librarians covered in a talk at the 2017 United Kingdom Serials Group Annual Meeting. Includes case studies related to seven named publishers and lists of specific issues raised.
- Highwire Press. Plan S: the options publishers are considering [Internet]. Highwire Press; 2019 [cited 12 Nov 2019]. <https://www.highwirepress.com/insight/plan-s-the-options-publishers-are-considering/>: A summary of the views of Highwire Press client journals about nine strategic approaches under consideration for implementing Plan S.
- Mayernik MS, Phillips J, Nienhouse E. Linking publications and data: challenges, trends, and opportunities. D-Lib Mag. 2016 May/Jun;22(5/6). DOI: http://dx.doi.org/10.1045/may2016-mayernik. A report on linking publication and data repositories, growing out of a National Science Foundation–sponsored conference held in early 2016.
- Sollenberger JF, Holloway Jr. RG. The evolving role and value of libraries and librarians in health care. JAMA. 2013 Sep 25;310(12):1231–2. DOI: http://dx.doi.org/10.1001/jama.2013.277050: An opinion piece on the roles librarians play in a variety of clinical settings and in providing consumer-oriented medical information.
- SPARC landscape analysis: The changing academic publishing industry – implications for academic institutions [Internet]. SPARC; 2019 [cited 12 Nov 2019]. <https://sparcopen.org/our-work/landscape-analysis/>: An in-depth market analysis of the major companies in two major publishing arenas: research and the higher education textbook market. Introductory sections (pages 6–9 for research; pages 35–40 for the higher education textbook market) lay out major issues.

## MLA INSIGHT INITIATIVE TASK FORCE

The task force is the steering committee for the multiyear InSight Initiative. The task force also reviews the applications from librarians expressing an interest to attend an InSight Initiative Summit and selects the participants based on the summit theme and participation of a representative mix of librarians affiliated with the diverse organizations with whom vendors work, including academic medical centers, community hospitals, specialty schools (nursing, pharmacy, etc.), governmental agencies, corporations, and nonprofit advocacy and community-based organizations.

Gerald J. Perry, AHIP, FMLA, University of Arizona, Chair, MLA Past President
Barbara A. Epstein, AHIP, FMLA, University of Pittsburgh, Member, MLA Past President
Michelle Kraft, AHIP, Cleveland Clinic Foundation, Member, MLA Past President
Gabriel R. Rios, Indiana University, Member
Daniel J. Doody, Doody Consulting, Summit Organizer
Rich Lampert, Doody Consulting, Summit Co-Organizer
Beverly Murphy, AHIP, FMLA, Duke University Medical Center, Board Liaison, MLA Past President
Kevin Baliozian, Medical Library Association, Member, MLA Executive Director
Mary M. Langman, Medical Library Association, Staff Liaison

## INSIGHT SUMMIT 3 PROGRAM COMMITTEE

The program committee developed the schedule and all program elements for InSight Initiative Summit 3. It was appointed by the InSight Initiative Task Force and consisted of three librarians, three representatives from participating organizations, the program facilitators, and a liaison from the InSight Initiative Task Force.

Daniel J. Doody, Doody Consulting, Summit Facilitator
Rich Lampert, Doody Consulting, Summit Facilitator
John Gallagher, Yale University, Member, Librarian Representative
Deborah Harris, F1000, Member, Industry Representative
David Nygren, American Psychological Association, Member, Industry Representative
Gerald (Jerry) Perry, AHIP, FMLA, University of Arizona, Liaison, InSight Initiative Task Force
Barbara Platts, AHIP, Munson Healthcare, Member, Librarian Representative
Amanda Sprochi, AHIP, University of Missouri–Columbia, Member, Librarian Representative
Michael Weitz, McGraw-Hill, Member, Industry Representative

## INSIGHT SUMMIT 3 FACILITATORS

Discussions and group exercises were facilitated by InSight Summit 3 Program Committee members.

Daniel J. Doody, Doody Consulting
Rich Lampert, Doody Consulting
Gerald (Jerry) Perry, AHIP, FMLA, University of Arizona

## INSIGHT SUMMIT 3 PARTICIPATING ORGANIZATIONS AND SPONSORS

MLA thanks the following participating organizations:

- American Psychiatric Association Publishing
- American Psychological Association
- American Society of Health-System Pharmacists (ASHP)
- BMJ Publishing Group
- F1000
- The JAMA Network
- Massachusetts Medical Society/NEJM
- McGraw-Hill Education
- Oxford University Press
- Springer Nature
- Wolters Kluwer Heath Learning Research & Practice

## INSIGHT SUMMIT 3 PARTICIPANTS

InSight Summit 3 had equal representation of librarian leaders and participating organizations.

Katherine G. Akers
  Wayne State University
Priya Arora
  Wolters Kluwer
Charlotte Beyer, AHIP
  Rosalind Franklin University of Medicine and Science
Saskia Bolore
  American Medical Association
John Gallagher
  Yale University
Karen Gutzman
  Northwestern University
Susan Haering
  Massachusetts Medical Society/NEJM Group
Deborah Harris
  F1000
Andrew Hickner
  Seton Hall University
Chris Jezowski
  American Society of Health-System Pharmacists (ASHP)
Lauren Jones
  BMJ
Andrea C. Kepsel, AHIP
  Michigan State University
Alisha Khan
  Wolters Kluwer Health
Elizabeth Laera, AHIP
  Princeton Baptist Medical Center
John McDuffie
  American Psychiatric Association
David Nygren
  American Psychological Association
Sean Pidgeon
  Oxford University Press
Barbara A. Platts, AHIP
  Munson Healthcare
Wyatt Reynolds
  Oxford University Press
Ryan Rodriguez
  BMJ
Rebecca Seger
  Oxford University Press
Jean Song
  University of Michigan
Angela Spencer, AHIP
  Saint Louis University
Amanda Sprochi, AHIP
  University of Missouri
Michael Weitz
  McGraw Hill Education
